# HEViTPose: towards high-accuracy and efficient 2D human pose estimation with cascaded group spatial reduction attention

**DOI:** 10.1038/s41598-026-35859-x

**Published:** 2026-01-17

**Authors:** Chengpeng Wu, Zhidong Chen, Beihua Ying, Guangxing Tan, Bing Hu, Chunyu Li, Haifeng Chen

**Affiliations:** 1 School of Cixi, Zhejiang Business Technology Institute, Ningbo, 315012 China; 2 College of Information and Intelligence Engineering, Zhejiang Wanli University, Ningbo, 315100 China; 3 College of Automation, Guangxi University of Science and Technology, Liuzhou, 545616 China; 4 College of Intelligent Manufacturing, GuangXi Vocational College of Safety Engineering, Nanning, 530100 China; 5 School of Electrical Engineering, Beijing Jiaotong University, Beijing, 100044 China

**Keywords:** Human pose estimation, Vision transformer, Attention, Efficient deep learning, Engineering, Mathematics and computing

## Abstract

Transformer-based human pose estimation methods have made encouraging progress in improving performance. However, the excellent performance of pose networks is often accompanied by heavy computational costs and large network scale. In order to deal with this problem, this paper proposes a High-accuracy and Efficient Vision Transformer for Human Pose Estimation (HEViTPose). Firstly, the concept of Patch Embedded Overlap Width (PEOW) is proposed to help understand the relationship between the amount of overlap and local continuity. By explicitly adjusting PEOW value, the model’s capacity to capture local continuity information is enhanced. Secondly, a Cascaded Group Spatial Reduction Multi-Head Attention (CGSR-MHA) is proposed, which improves memory efficiency through feature grouping, reduces computational cost through spatial reduction, and also improves network performance by retaining multiple low-dimensional attention heads. Finally, comprehensive experiments on two benchmark datasets (MPII and COCO) demonstrate that the HEViTPose model performs on par with the state-of-the-art models, but is more lightweight while possessing higher inference speed. Specifically, compared with HRNet with similar performance and inference speed, the proposed model reduces the number of parameters by 62.1% and the amount of computation by 43.4%. Compared with HRFormer with similar performance and network size, the inference speed is about 2.6 times faster. Code and models are available at https://github.com/ T1sweet/HEViTPose.

## Introduction

Human pose estimation (HPE) is a fundamental research topic in the field of computer vision and plays a crucial role in human-centred vision applications. The goal of HPE is to localize the exact pixel positions of keypoints of body parts from the images by detection and estimation methods. However, the development of HPE faces significant challenges when dealing with complicated situations, including viewpoint and appearance variations, occlusion, multiple persons, and imaging artefacts, etc. With further research, HPE has a wide range of applications in action recognition^1^, human-robot interaction^2^, intelligent surveillance^3^, intention recognition^4^, and automated driving^5^, as one of the fundamental tasks of understanding human behaviour.

Developments in recent years have shown that deep learning-based methods have achieved state-of-the-art results in solving the Human pose estimation problem. There are currently two mainstream methods: (i) first predicting keypoints heatmap and then converting them to position^6–9^, and (ii) directly regressing keypoints position^10–12^. In this paper, we adopt the heatmap-based paradigm, which is widely recognized for its exceptional advantage in keypoint localization accuracy. Although this approach is generally more computationally intensive than regression-based methods, we argue that its computational demand can be effectively mitigated through a compact and efficient model design, thereby striking an optimal balance between localization precision and inference efficiency. The typical architecture consists of a backbone network for feature extraction and a regressor for heatmap estimation.

According to the form of the different combinations of the backbone networks and the regressors, different types of network architectures are extended. A common used network framework is to connect from high resolution to low resolution and then from low resolution to high resolution, such as SimpleBaseline^6^, Hourglass^7^. Another network framework maintains high resolution throughout the process by connecting multi-resolution subnets in parallel, such as HRNet^9^ and its variants^13,14^. In addition, multi-scale fusion^8,15,16^ and multi-stage supervision^17^ can be integrated into both types of network frameworks. For intensive prediction tasks such as HPE, the ability of the backbone network to extract features often determines the performance of the model. Therefore, this paper simply follows the network architecture of SimpleBaseline^6^ and focuses on the design of the backbone network.

**Fig. 1:**
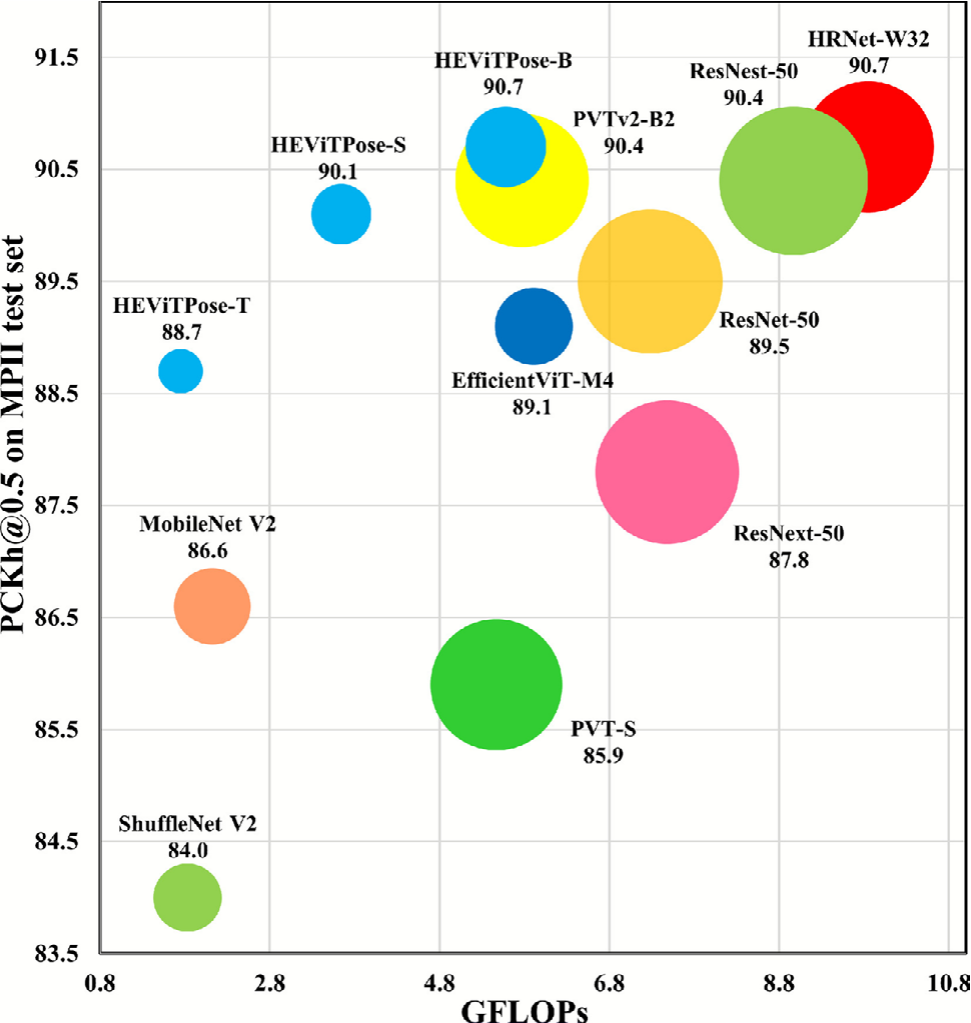
Comparison of HEViTPose and SOTA network models on the MPII test set regarding performance, parameters, and GFLOPs. The size of each bubble represents parameters.

The improvement of the performance of backbone networks mainly relies on the development of feature extraction techniques. For a long time, Convolutional Neural Network (CNN) have achieved remarkable success in computer vision due to their excellent feature extraction capability, becoming the dominant method in this field, such as^7,9,18–20^. However, the feature extraction capability of CNN is restricted by the receptive field. In order to extract long-range features, the receptive field has to be expanded by increasing the depth of the network, even if the feature contains only a small amount of information, which results in a larger network size and a higher computational overhead. As a result, the CNN-based network model shows a significant increase in parameters and GFLOPs with gradual improvement in performance, such as MobileNetV2^20^, ResNet-50^18^, and HRNet-W32^9^ in Fig. 1.

Recently, there has been a number of Transformer-based backbone networks proposed, which have received a lot of attention in the field of computer vision due to their excellent long-distance modelling capability and outstanding performance, such as^21–25^. The Transformer-based model outperforms the classical CNN-based model on large datasets. However, when the amount of data is insufficient, the Transformer-based model will fall behind due to the difficulty in exploiting its powerful feature extraction capability. As shown in Fig. 1, PVTv2-B2^26^ slightly underperforms HRNet-W32^9^ of the same size in terms of performance on the MPII test set. It is worth noting that by adopting the Transformer-based approach we should take advantage of its long-range modelling capability rather than relying on a large number of block stacks.

In this study, we design the network architecture of HEViTPose for HPE tasks by taking inspiration from established networks (such as EfficientViT^25^, Swin^24^, PVT^22^, and PVTv2^26^) to ensure a balance between model performance, size and computational overhead, as shown in Fig. 3. The HEViTPose shows a well-balanced performance in all aspects and surpasses all models shown in Fig. 1. The main contributions of this work are summarized as:

- We introduce the concept of PEOW based on OPE^26^, further reveal the relationship between the number of overlapping edges and local continuity through experiments, and gradually improve the indicators of the model through the optimisation of PEOW.
- We propose a CGSR-MHA module that combines the benefits of CGA^25^, SRA^22^, and MHA^27^. This module significantly decreases computational costs by incorporating feature grouping and spatial degradation mechanisms, while maintaining feature diversity with multiple low-dimensional attention heads.
- Through experimental validation on two benchmark datasets, the Human pose estimation algorithm designed in this paper is found to be much improved compared to other mainstream algorithms. Specifically, HEViTPose achieves an average accuracy of 90.7% and 72.6% on the MPII^28^ and COCO^29^ test datasets, respectively, which possesses a lower number of parameters and computation amount compared to network models with similar performance, as well as faster inference speed, which makes it more competitive compared to mainstream lightweight Human pose estimation algorithms.

## Related work

### Human pose estimation

The algorithmic frameworks for 2D multi-person pose estimation are classified into top-down^6,8,9,30^ and button-up^31–34^. The top-down algorithmic framework has been decomposing the multi-person pose estimation task into two sub-tasks, multi-person detection^35–37^ and single-person pose estimation, which is considered to be high in accuracy, high in computation and slow in inference. While button-up algorithmic framework decomposes the task into two subtasks of keypoint detection and keypoint grouping for multiple people, which is considered to be computationally fast and less accurate. In recent years, the continuous advancement of object detection algorithms^38–40^ has led to the promotion of the top-down algorithm framework, making a significant breakthrough in inference speed. As a result, it has gradually emerged in the task of real-time human pose estimation. This paper conducts research on HPE via a top-down algorithmic framework, primarily concentrating on the architectural design of the backbone network.

### Transformer based vision backbones

The study of backbone architectures in this paper is an extension of ViT^21^ and its related studies^22–24,41^. ViT^21^ divides images into medium-sized image blocks and converts them into a series of fixed-length patch embeddings, and performs image classification through the Transformer architecture, achieving a balance between speed and accuracy. However, the excellent performance of ViT relies heavily on the support of large-scale training datasets. In order to address this issue, DeiT^41^ outlines various training approaches and distillation techniques that enhance data efficiency, thus rendering ViT more efficient when dealing with smaller datasets. Several studies in the same period provided ideas to improve the performance of Transformer-based network architectures. PVT^22^ introduces a pyramid structure to construct a multi-resolution feature map, which achieves better accuracy in dense prediction tasks. LocalViT^42^ incorporates depth-wise convolution into ViT to improve the local continuity of features. Swin^24^ adopts local window self-attention instead of global self-attention, reducing the quadratic relationship between network complexity and image size to a linear relationship and achieving a speed-accuracy balance. In addition, MHSA^27^ embeds the input features into multiple subspaces by attention head number and computes the attention maps separately, which has been shown to help improve model performance. However, improving performance simply by increasing the number of heads of attention is inefficient and creates significant computational redundancy. The work of EfficientViT^25^ shows that assigning different splits of the complete feature to different attention heads can effectively reduce attentional computational redundancy. This problem-solving approach follows the same line of thought as grouping convolution^43,44^. In order to prevent the performance loss caused by excessive grouping, this work has employed two strategies. Firstly, it has appropriately increased the number of attention heads within the group. Secondly, it has controlled the dimensions of the Q, K, and V projections to correspond with the number of heads. This approach can significantly reduce the computational overhead while ensuring the network performance.

### High resolution feature maps

The programme of high-resolution feature maps has been a great success on the HPE mission. In the development of high-resolution feature maps, four main approaches have emerged, including: (i) Dilated convolutions^45,46^ maintain the high resolution of the feature map by removing some downsampling layers, preventing the loss of spatial information but incurring more computational cost. (ii) Stacked Hourglass^7^, CPN^8^ utilise a decoder to restore high-resolution representations from low-resolution representations. (iii) The high-resolution representation of the HRNet^9^ model consists of different subnetworks with different resolutions, ensuring that the network retains its high resolution, and generates high-resolution feature maps with rich information through multi-scale fusion between branches. (iv) Transposed convolution^6,16^ improves the resolution of the feature maps at the end of the network. SimpleBaselines^6^ demonstrates that transposed convolution can generate high-quality feature maps for heatmap prediction. Our proposed HEViTPose follows the SimpleBaselines^6^ approach to generate high-resolution feature maps from low-resolution feature maps extracted from the backbone network by transposed convolution.

## Method

### Overall architecture

For the HPE task, a network model called HEViTPose is designed in this paper, as shown in Fig. 3a. In the patch embedding part of the input image, we are inspired by the Overlapping Patch Embedding (OPE)^26^ to propose a concept of Patch Embedding Overlap Width (PEOW), and design an optimisation experiment of PEOW in Sec. 4 to help readers further understand the relationship between the amount of overlap and local continuity. In the backbone network part, we implement the design concept of PVT^22^, which involves the incorporation of the progressive pyramid structure^15^ into the Transformer framework. This enhances the performance of the HPE task by generating multi-scale feature maps. We have meticulously designed the Transformer-based backbone network also called HEViTPose. The network consists of three stages, each with a similar architecture, including a patch embedding layer, a downsampling layer (except for the first stage), and a transformer coding layer. When presented with an input image of size $H\times W\times 3$, the network generates three feature maps sequentially, producing a feature pyramid of $\frac{H}{4} \times \frac{W}{4} \times C_1$, $\frac{H}{8} \times \frac{W}{8} \times C_2$, and $\frac{H}{16} \times \frac{W}{16} \times C_3$. In the head network part, we directly perform two up-sampling (transposed convolution) operations on the feature maps extracted from the backbone network, and the generated high-resolution feature maps ($\frac{H}{4} \times \frac{W}{4} \times 16$)^6^. In the regressor section, we simply regress the 16 keypoint heatmaps with $\frac{H}{4} \times \frac{W}{4} \times 16$ feature maps generated by the head network, while defining the loss function as the mean square error of the predicted heatmap and the groundtruth heatmap. Here the groundtruth heatmap is generated by a 2D Gaussian algorithm with a standard deviation of 1 pixel centred on the groundtruth position of each keypoint.

### Patch embedding overlap width

**Fig. 2:**
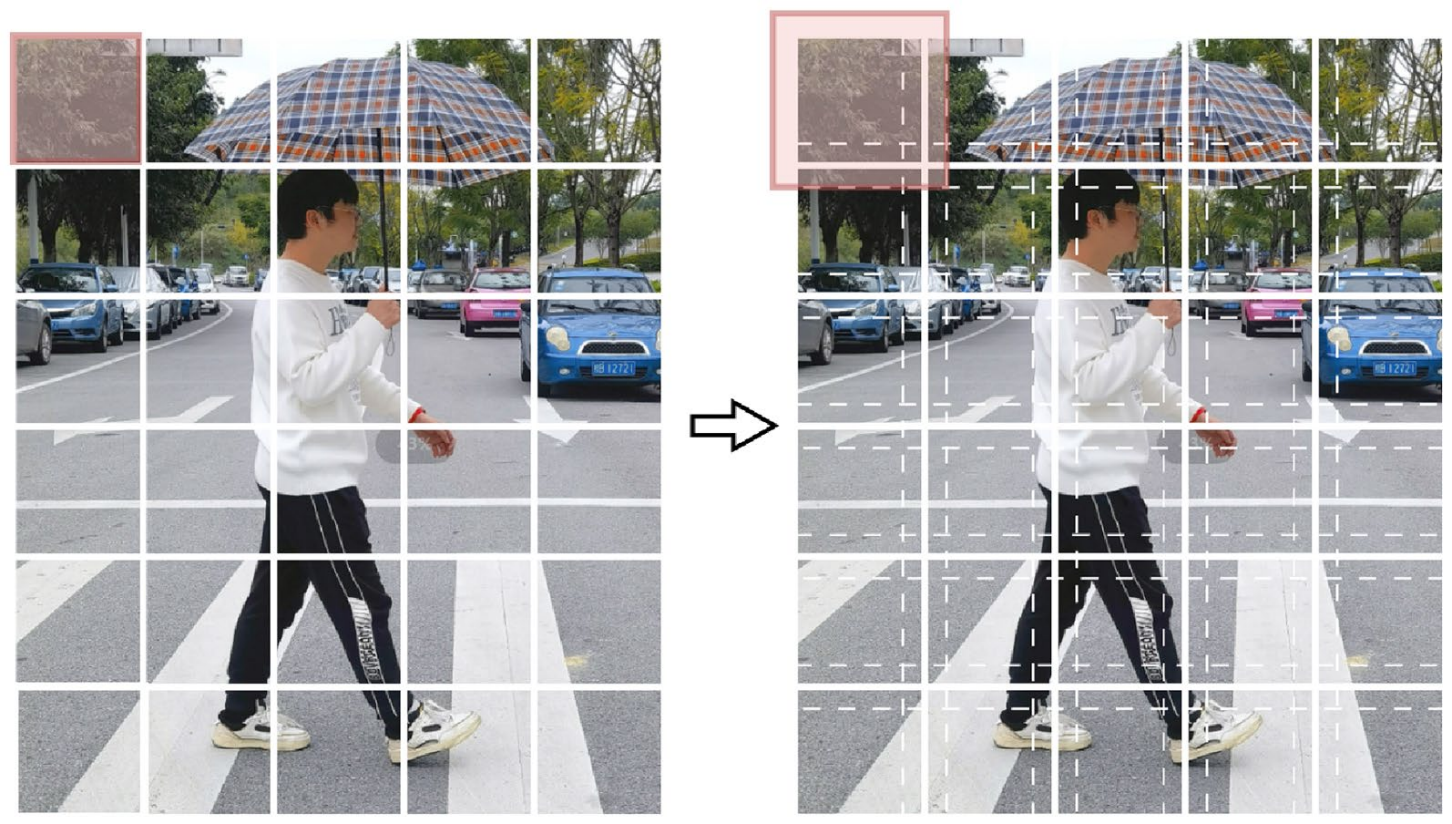
Left: Patch Embedding of ViT; right: Overlapping Patch Embedding of PVTv2. The image block under the pink mask represents the first patch of each of these two methods.

**Fig. 3:**
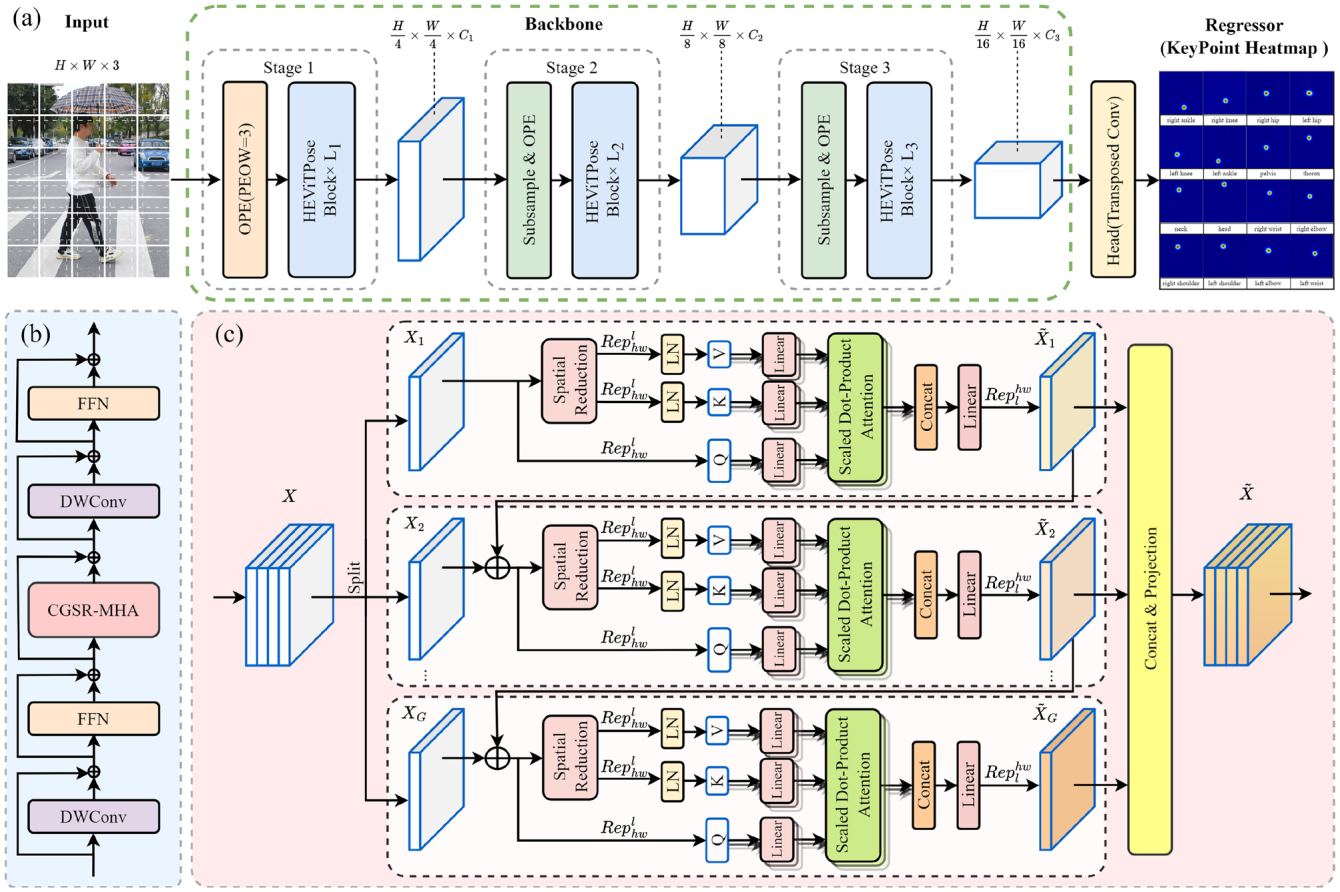
Overview of HEViTPose. (**a**) Network Architecture of HEViTPose; (**b**) HEViTPose Block; (**c**) CGSR-MHA.

ViT^21^ directly divides the image into non-overlapping patches, and then extracts embedding features for different patches separately through the Transformer network architecture, as shown in the left part of Fig. 2. However, truncating the image causes a loss of continuity information between patches, making it challenging to reconstruct all continuity information even when combining various embedding features. PVTv2^26^ preserves the local continuity of the image by extracting features through OPE, resulting in an improvement in the performance of the network, as shown in the right part of Fig. 2. Since the work of PVTv2 did not provide a detailed analysis of image patch overlap and local continuity, this section builds on OPE to portray the relationship between the amount of image patch overlap and local continuity through the relationship between the amount of image patch overlap and network performance (Fig. 3).

#### Definition of PEOW

**Fig. 4:**
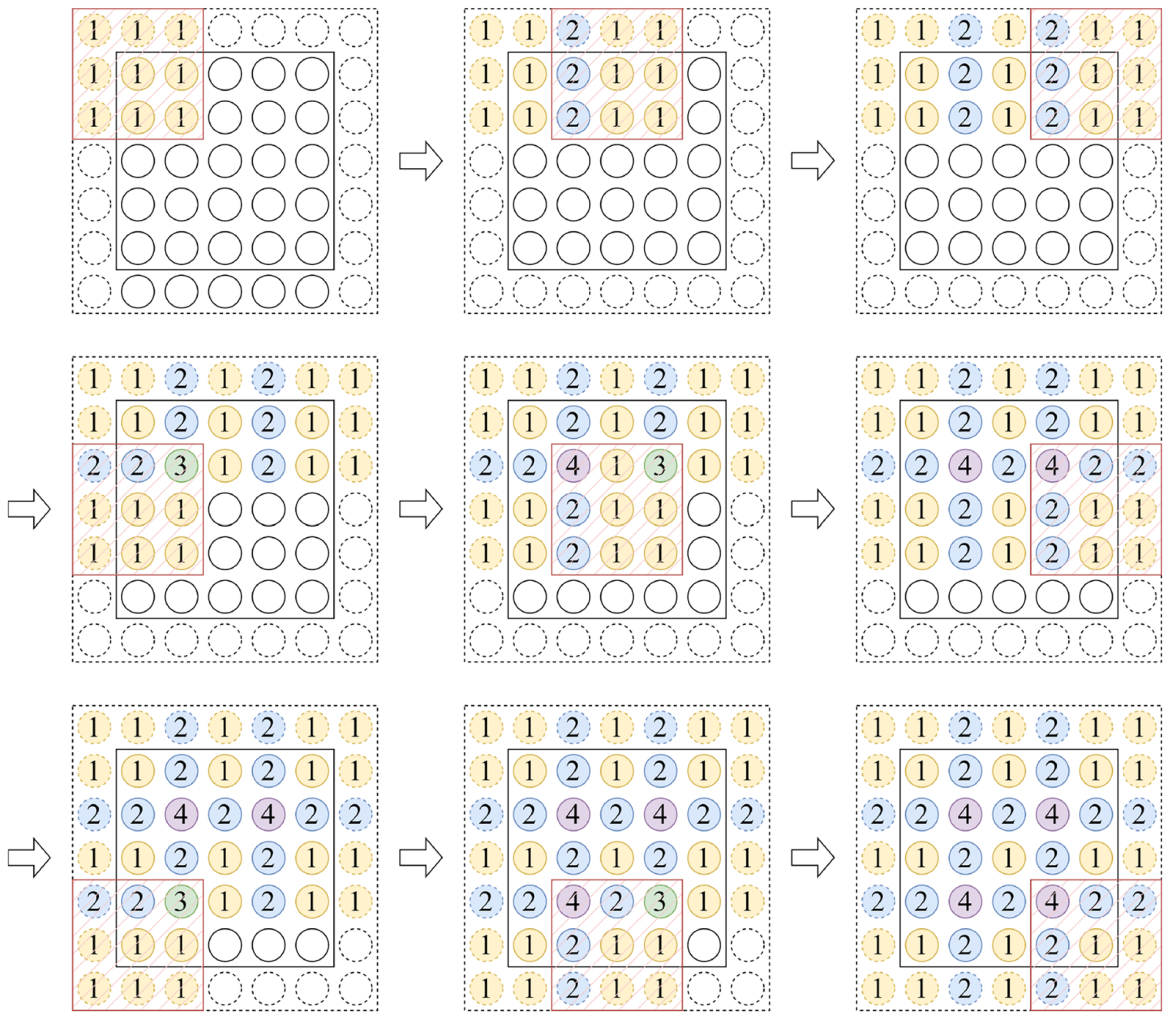
Set the convolution operation Conv2d(3, 2, 1) corresponding to PEOW=1. Where solid circles indicate image pixels, dashed circles indicate padding pixels, and the 3$\times$3 mask indicates the convolution kernel.

To quantify the impact of image patch overlap on local continuity, we define a tunable parameter, the ’Patch Embedding Overlap Width’, based on the Overlapping Patch Embedding (OPE) mechanism. This parameter is numerically equal to the difference between the convolutional kernel size and the stride (PEOW = kernel size - stride). It directly controls the pixel width of the overlapping region and serves as a more intuitive design variable than the ’overlapping ratio’. Figure 4 shows the case of PEOW = 1, where the number in the circle indicates the number of times an image pixel is used for computation when it is convolved.

#### Analysis

By observing the sliding process of the convolution kernel in Fig. 4. we find that: (i) A pixel with number 1 can be associated with elements in 1 region covered by the convolution kernel; (ii) A pixel with the number 2 can be associated with elements in 2 regions covered by the convolution kernel; (iii) A pixel with a number of 4 may be associated with elements in 4 regions covered by the convolution kernel.

The modeling process of a convolution kernel is shown in Fig. 6. The continuity between two adjacent image patches can be modeled through three pixel point samples. The continuity between four pairwise adjacent image patches can only be modeled by a sample of 1 pixel point.

Assuming that the modelling function $\mathcal {F}(\cdot )$ in this layer contains operations such as convolution, activation function $act(\cdot )$, etc., the predicted values of the four outputs in Figure 6 are $\hat{y}_1$, $\hat{y}_2$, $\hat{y}_4$, $\hat{y}_5$, as in Eqs. 1, 2, 3 and 4. Assuming that the overall modelling function is $\mathcal {G}(\cdot )$, the final output of any of the predicted values is $\hat{z}_i$, as in Eq. 5.

1 $$\begin{aligned} \hat{y}_1&= act(x_{11}w_{11}+ \cdots +x_{33} w_{33} ) \nonumber \\&= \mathcal {F}(x_{13}, x_{23}, x_{31}, x_{32}, x_{33}) \end{aligned}$$

2 $$\begin{aligned} \hat{y}_2&= \mathcal {F}(x_{13}, x_{23}, x_{33}, x_{34}, x_{35}) \end{aligned}$$

3 $$\begin{aligned} \hat{y}_4&= \mathcal {F}(x_{31}, x_{32}, x_{33}, x_{43}, x_{53}) \end{aligned}$$

4 $$\begin{aligned} \hat{y}_5&= \mathcal {F}(x_{33}, x_{34}, x_{35}, x_{43}, x_{53}) \end{aligned}$$

5 $$\begin{aligned} \hat{z}_i&= \mathcal {G}(\hat{y}_1,\hat{y}_2,...,\hat{y}_4,\hat{y}_5,...) \end{aligned}$$

We can observe from Eqs. 1, 2, 3, 4 and 5 that although the deep model $\mathcal {G}$ has all the input variables, it is relatively difficult to establish continuity through different layers of variables nested in functions. However, we can provide a wealth of information for building local continuity by controlling PEOW in the shallow model $\mathcal {F}$. Thus, two points for further consideration arise. (i) Although PEOW=1 can provide local continuity information, the shallow model is limited in its ability to capture continuity due to the small amount of information provided. (ii) For models supervised by Gaussian heatmaps, the derivative information around the mean is also an important part of what constitutes an accurate prediction, which cannot be modelled by PEOW=1.

**Fig. 5:**
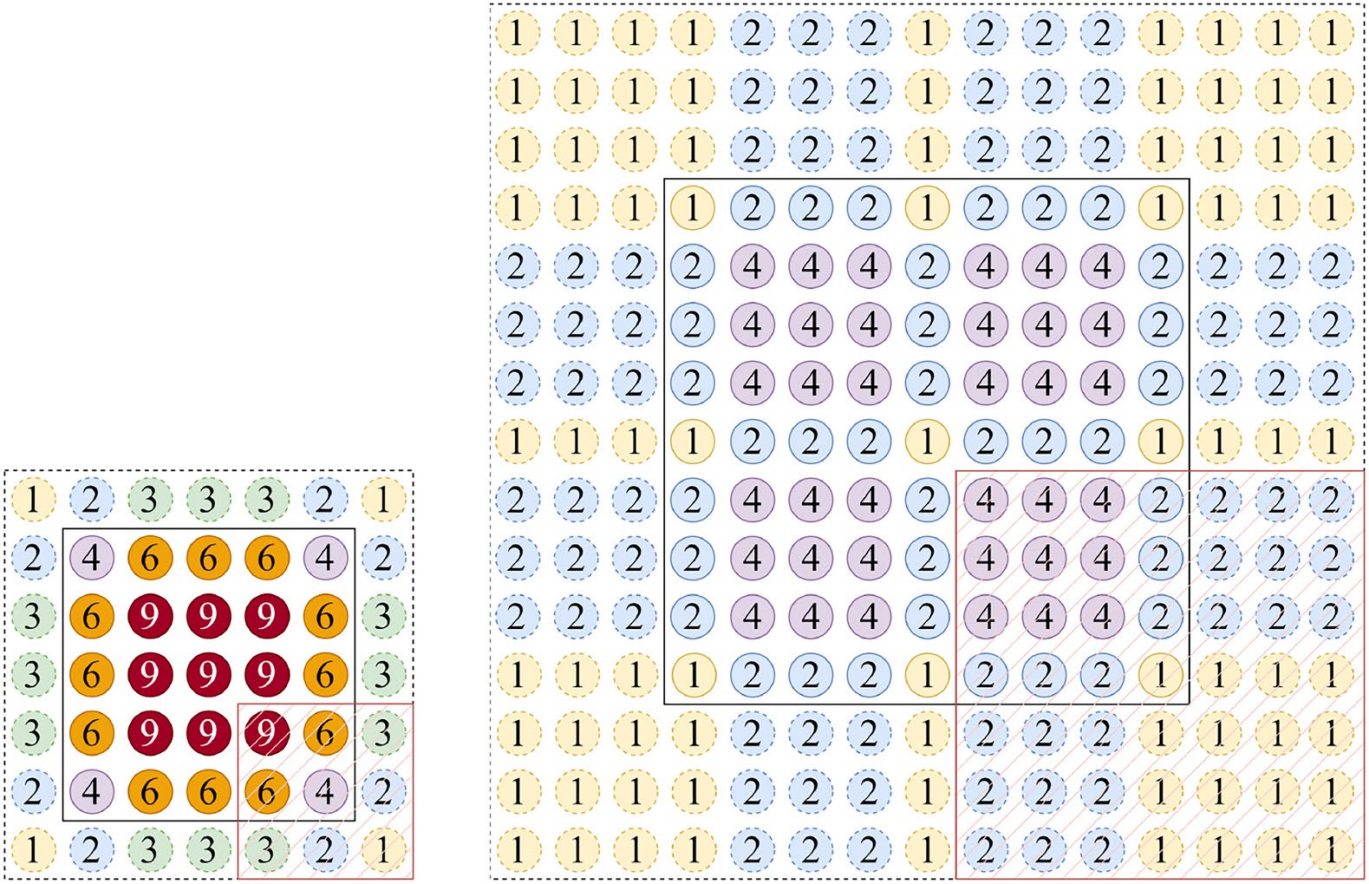
Two ways to increase the PEOW value. (**a**) Left: increasing the PEOW value by decreasing the stride (Conv2d(3,1,1)); (**b**) right: increasing the PEOW value by increasing the convolution kernel (Conv2d(7,4,3)).

Following this idea, we propose to supplement the continuity and conductibility information by adding PEOW solutions. We do not analyse the method of increasing the PEOW value by decreasing the stride, because this method will make the adjacent regions appear multiple similar computations, which is tend to produce information redundancy, as shown in Fig. 5a. Therefore, we use the method of increasing the size of convolutional kernel to increase PEOW, as shown in Fig. 5b. In the experimental section, we select different PEOWs to conduct experiments to verify the reasonableness of the thinking.

### HEViTPose building block

In order to achieve a balance between model performance and feature extraction efficiency, we first introduce a sandwich layout from EfficientViT^25^ in the HEViTPose building blocks to reduce the memory time consumption caused by the self-attentive layer in the model and to enhance communication between channels. Secondly, we follow the PVTv2^26^ approach by replacing the fixed-size positional embedding with the positional coding introduced by the zero-padded convolutional layer (DWConv) to adapt to arbitrary size input images, as shown in Fig. 3b. Thirdly, we have utilised the successful practices of MHSA^27^, CGA^25^, SRA^22^, and other attentional mechanisms to propose a Cascaded Group Spatial Reduction Multiple Head Attention (CGSR-MHA), which is more accurate in terms of precision, more computationally efficient, and more suitable for such intensive tasks as human pose estimation, by providing each subgroup with a different segmentation of the full feature and thus explicitly decomposing the attentional computation between subgroups, as in Fig. 3c.

#### CGSR-MHA

The Cascaded Group Attention (CGA) proposed by EfficientViT^25^ alleviates the attention head redundancy problem of Multiple Head Self-Attention (MHSA), resulting in an improvement in feature extraction efficiency. However, the CGA method exclusively divides each cascade group feature differently into Q, K, and V matrices for ablation experiments, which is more conservative in solving the information redundancy problem. Therefore, this paper adopts the idea of PVT^22^ to reduce the dimensionality of the features within the cascade group, and solves the information redundancy problem by directly controlling the dimensionality of the Q, K, V matrices. Formally, CGSR-MHA can be formulated as:

We divide the input feature $X\in \mathbb {R}^{C\times H \times W}$ into *G* groups: $X=[X_1,X_2,...,X_G]$, where $X_g\in \mathbb {R}^{\frac{C}{G}\times H\times W}$ denotes the feature map for each cascade grouping and $g\in [1,2,...,G]$ denotes the number of each cascade grouping.

6 $$\begin{aligned} \tilde{X}_{g} =\left\{ \begin{aligned} \mathcal {L}(X_g, r), g&= 1 \\ \mathcal {L}(X_{g}+\tilde{X}_{g-1}, r), g&\in \left( 2,3,\ldots ,G \right) \end{aligned} \right. \end{aligned}$$

where $\mathcal {L}(\cdot )$ denotes the attention function within the cascade group, and $X_g \in \mathbb {R}^{\frac{C}{G}\times H\times W}$ denotes the feature map within the cascade group after $\mathcal {L}(\cdot )$ processing. The specific operation process of $L(X_g,r)$ is as follows:

7 $$\begin{aligned}&Q={Rep\,}_{{hw}}^{{l}}({{X}}_{{g}}) \end{aligned}$$

8 $$\begin{aligned}&K,V=LN({Rep\,}_{{hw}}^{{l}}(SR({{X}}_{{g}},r))) \end{aligned}$$

where $Rep_{hw}^l(\cdot )$ denotes the operation of reshaping the features in the group of size $\frac{C}{G} \times H \times W$ into $\frac{C}{G} \times L$, and $L= HW$ denotes the encoding length. $SR(\cdot )$ denotes the spatial reduction operation, *r* denotes the spatial reduction rate, and the size of the feature map obtained after spatial reduction of $X_g$ is $\frac{C}{G} \times \frac{H}{r} \times \frac{W}{r}$. $LN(\cdot )$ denotes the layer normalization operation. The matrices $Q \in R^{\frac{C}{G} \times HW}$, $K,V \in R^{\frac{C}{G} \times \frac{HW}{r^2}}$.

9 $$\begin{aligned}&\tilde{X}_{1}={Rep\,} _{{l}}^{{hw}}(MHA(Q,K,V)) \end{aligned}$$

10 $$\begin{aligned}&\tilde{X}=Linear(Concat(\tilde{X}_{1},\tilde{X}_{2},...,\tilde{X}_{G})) \end{aligned}$$

where $Rep_{l}^{hw}(\cdot )$ denotes the operation of reshaping a feature of size $\frac{C}{G} \times L$ to $\frac{C}{G} \times H \times W$. $MHA(\cdot )$ denotes the multi-head attention operation. $Linear(\cdot )$ denotes the linear projection operation, $Concat(\cdot )$ denotes the splicing operation, and $\tilde{X}$ denotes the feature map obtained after the input feature map *X* is subjected to the CGSR-MHA($\cdot$) operation.

Different from the spatial reduction strategy used by CGA^25^, CGSR-MHA can achieve linear computation and memory cost like convolutional layers by reasonably controlling the number of groupings *G* and spatial reductions *r*. At the same time, by paying reasonable attention to the number of heads *h*, it can effectively improve the model performance. Specifically, given an input of size $H\times W\times C$, the complexity of CGA and CGSR-MHA are, respectively.

11 $$\begin{aligned} \begin{aligned} \Omega \text {(CGA)}=(2dC+\frac{C^2}{G}+K^2 dG)HW\\ +(C+dG) M^2 HW+HWC^2 \end{aligned} \end{aligned}$$

12 $$\begin{aligned} \begin{aligned} \Omega \text {(CGSR-MHA)}=(\frac{C^2}{G}+\frac{2C^2}{Gr^2})HW\\ +\frac{2H^2 W^2 C}{r^2} +HWC^2 \end{aligned} \end{aligned}$$

where the hyperparameters in CGA are $d=16$, $G=4$, $K=5$, and $M=7$. The hyperparameters in CGSR-MHA are determined through the experiments in Sec 4, with the aim of achieving a balance between the model in terms of performance, inference efficiency, and computational overhead by selecting a suitable set of hyperparameters.

#### Model families of HEViTPose

In order to compare the network models of different scale, we build a model family containing three models and show the details of the architecture of each model in Table 1. $C_i$, $L_i$, $G_i$ are the width, depth and number of groupings of the ith stage, respectively.

**Table 1:** Architectural details of the HEViTPose model family.

Model	$\{C_1,C_2,C_3\}$	$\{L_1,L_2,L_3\}$	$\{G_1,G_2,G_3\}$
HEViTPose-T	{64,128,192}	{1,2,3}	{4,4,4}
HEViTPose-S	{128,192,224}	{1,2,3}	{4,3,2}
HEViTPose-B	{128,256,384}	{1,2,3}	{4,4,4}

## Experiments

### MPII human pose estimation

#### Dataset

The MPII Human Pose dataset^51^ consists of approximately 25k images from a wide range of real-world activities, containing 40k person instances and full-body pose annotations marked with 16 key points. The dataset has around 28k person instances used as training samples and around 12k person instances used as test samples.

#### Training details

In this paper, we follow the common training strategy of the mmpose codebase^51^, setting up different data pipelines for the training set and the validation set.

For the training set, our cropping strategy is to first extend the human detection box in height or width to a fixed aspect ratio (height:width = 1.25), and then random shift with a shift factor (0.16). Then, we design a data augmentation strategy that includes random flipping, random half body transformation^52^, random rotation $([-40^{\circ }, 40^{\circ }])$, and random scaling ([0.5,1.5]), with the main purpose of learning scale invariance and rotation invariance. Finally, we use affine transformations to adjust the image to a fixed size $(256 \times 256)$ as input to the network.

For the validation set, we simply extend the human detection box to a fixed aspect ratio (height:width = 1.25) and then use affine transformations to adjust the image to a fixed size $(256 \times 256)$ as input to the network.

The model was trained on NVIDIA RTX3060 GPU (12GB). We use the Adam^53^ optimizer. The learning schedule follows the setting^54^. The base learning rate is set to 1e-3, and dropped to 1e-4 and 1e-5 at the 170th and 200th epochs, respectively. The training process is terminated within 210 epochs. Due to GPU memory limitations, we set the training batch size to 32 for all models to ensure that all models could be compared fairly. Note that all models are trained from scratch without any pre-training.

#### Testing details

We follow the two-stage top-down multiple human pose estimation paradigm similar as^8,54^, which consists of using object detectors to detect human instances and using pose estimation networks to generate keypoint predictions for the instances. We use the same person detectors provided by SimpleBaseline^6^ for both the validation set and the test set. Following^7,8^, we first compute the heatmap by averaging the heatmap of the original image and the flipped image, and then convert the predicted heatmap into position coordinates using the standard decoding method. The decoding method first shifts the highest response position of the key point prediction heat map to the second highest response position by 1/4, and then directly converts the offset position into coordinates as the final prediction result.

#### Results on the validation set and test set

In this section, we compare HEViTPose with several representative convolutional methods (including HRNet^9^, hourglass^7^), Transformer methods (including ViTPose^49^, PVTv2^26^, Swin^24^), and hybrid convolutional and Transformer methods (such as HRFormer^23^). Tables 2 and 4 report the model PCKh@0.5 scores, the number of model parameters (Params) and model operations (FLOPs) on the MPII validation and test sets, respectively. Table 3 reports the inference speeds of models with similar performance.

**Table 2:** Comparison on the MPII validation set (PCKh@0.5). The performance, parameters, and GFLOPs for the pose estimation network are measured w/o considering human detection. All results come from retraining under the same conditions, and none uses any pre-training.

Method	Params	FLOPs	Hea	Sho	Elb	Wri	Hip	Kne	Ank	Total
MobileNetV2^20^	9.57M	2.12G	95.6	93.8	84.8	77.8	85.7	79.4	73.0	85.0
Hourglass-52^7^	94.85M	28.67G	96.5	95.5	88.8	83.8	88.2	85.0	80.8	88.9
ResNest-50^47^	35.93M	8.97G	96.3	95.6	89.1	84.0	87.8	84.9	80.4	88.8
ResNet-50^18^	34.0M	7.28G	96.1	95.1	88.2	81.9	88.1	83.0	77.5	87.7
ResNext-50^48^	33.47M	7.48G	95.8	95.0	88.0	82.5	88.1	83.2	77.5	87.8
HRNet-W32^9^	28.02M	9.85G	96.8	95.6	89.9	85.5	89.3	85.3	81.5	89.6
ViTPose-B^49^	90.04M	23.8G	95.2	92.8	84.5	78.8	85.4	80.2	74.8	85.2
PVT-S^22^	28.17M	5.47G	95.0	93.3	84.0	77.4	85.0	78.1	72.8	84.4
Swin-S^24^	54.1M	15.4G	96.1	94.8	87.2	80.8	87.9	82.3	77.8	87.3
EfficientViT-M4^25^	9.87M	5.91G	95.9	94.8	87.4	81.7	87.9	83.4	78.2	87.6
HRFormer-S^23^	7.53M	3.52G	96.2	94.6	88.7	83.3	88.1	83.5	78.4	88.1
PVTv2-B2^26^	29.05M	5.77G	96.5	95.9	89.0	83.6	89.2	84.2	79.5	88.9
HEViTPose-T	3.21M	1.75G	95.9	94.9	87.4	81.6	87.4	81.6	77.2	87.2
HEViTPose-S	5.88M	3.64G	96.3	95.2	88.7	83.3	88.5	83.9	79.5	88.5
HEViTPose-B	10.63M	5.58G	96.5	95.6	89.5	84.5	89.1	85.7	81.1	89.4

**Table 3:** The parameters, computation, inference speed and performance of the model are compared on the MPII and COCO validation sets.

Method	GPU (FPS)	CPU (FPS)	Params	FLOPs	MPII (Total)	COCO(AP)
HRFormer-B^23^	8.1	0.5	40.04M	17.35G	90.2	77.0
HRFormer-S^23^	15.8	1.9	7.53M	3.52G	88.1	73.4
HRNet-W32^9^	31.9	2.5	28.02M	9.85G	89.6	76.1
Hourglass-52^7^	25.7	1.3	94.85M	28.67G	88.9	–
EfficientViT-M4^25^	37.6	2.6	9.87M	5.91G	87.6	–
MobileNetV2^20^	67.9	6.2	9.75M	2.12G	85.0	58.9
MobileNetV3^50^	78.5	7.1	15.46M	1.73G	84.4	62.2
ShuffleNetV2^19^	75.2	8.5	7.55M	1.83G	82.4	60.2
LiteHRNet-30^13^	29.9	4.5	1.76M	0.56G	85.1	61.5
PVTv2-B0^26^	28.5	2.1	29.05M	5.77G	88.9	75.7
HEViTPose-B	34.5	2.7	10.63M	5.58G	89.4	75.4
HEViTPose-S	41.3	3.3	5.88M	3.64G	88.5	–
HEViTPose-T	47.4	5.4	3.21M	1.75G	87.2	–

**Fig. 6:**
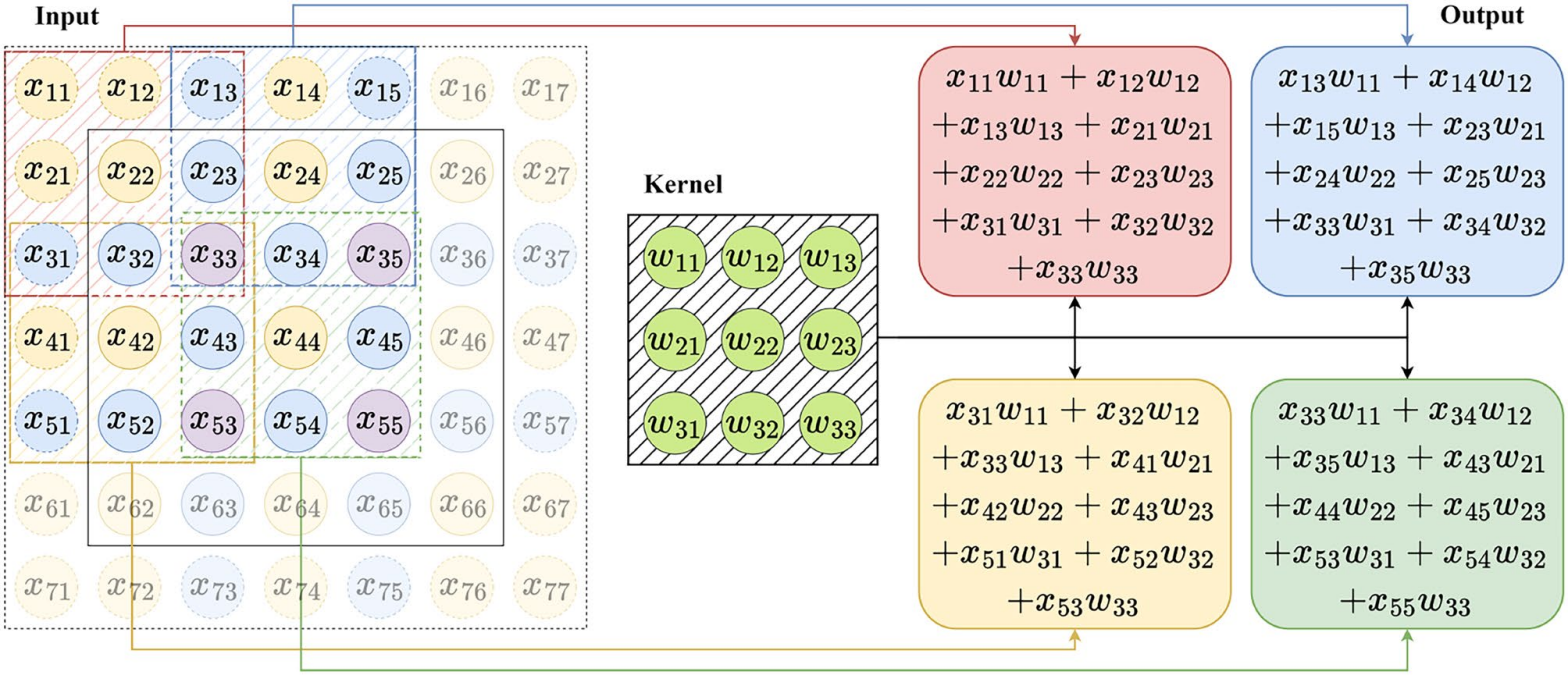
Modelling process for convolution with PEOW = 1. For ease of understanding, here let the bias b = 0.

The HEViTPose model is trained from zero with an input size of $256 \times 256$ and achieves a PCKh@0.5 score of 89.4 on the MPII validation set. Compared with other models with similar performance, HEViTPose has a large advantage in terms of reduced model size and computation, as shown in Table 2. For example, (i) compared with the convolutional models with comparable performance, HRNet-W32^9^ and Hourglass-52^7^, HEViTPose-B has 62.1% and 88.8% fewer Params and 43.4% and 80.5% fewer FLOPs, respectively. (ii) Compared with the Transformer model PVTv2-B2^26^, HEViTPose-B has 63.4% fewer Params with similar performance and computation (Fig. 6).

**Fig. 7:**
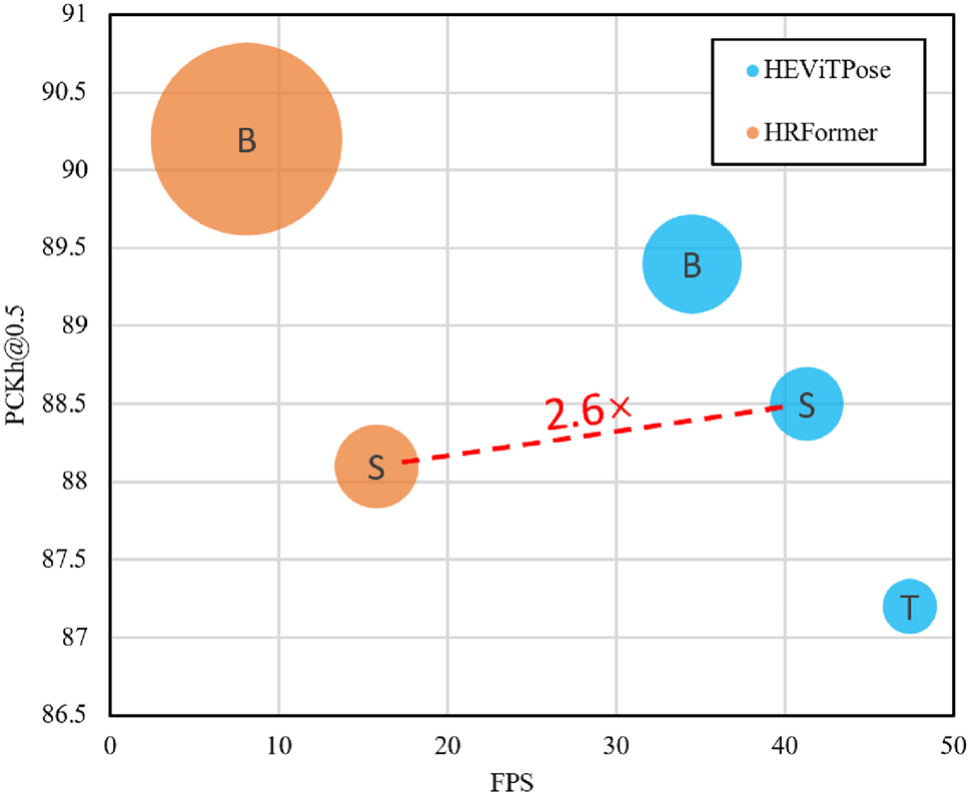
Inference speed comparison of performance similar models. The size of each bubble represents the number of model parameters.

HEViTPose is also more competitive in terms of inference speed compared to other models with similar performance, as shown in Table 3. In particular, compared to the hybrid convolutional and Transformer model HRFormer-S^23^, HEViTPose-S has 21.9% fewer Params, similar performance and computation, and 2.6 times faster inference on GPU, as in Fig. 7. Furthermore, compared with the lightweight model LiteHRNet-30^13^, although the parameter quantity of HEViTPose-T increased by 1.45M and the computing power increased by 1.19GFLOPs, the PCKh@0.5 score improved by 1.9, the inference speed on GPU increased by 17.5FPS, and the inference speed on CPU increased by 0.9FPS. Even when compared with the inference model MobileNetV2^20^, although the inference speed of HEViTPose-T on GPU decreased by 20.5FPS and on CPU decreased by 0.8FPS, the parameter quantity decreased by 66.5%, the computing power decreased by 17.5%, and the PCKh@0.5 score increased by 2.1. Finally, compared to PVTv2-B0^26^, our HEViTPose-B achieves a 0.5 higher PCKh@0.5 score on the smaller MPII dataset but shows a 0.3 lower AP score on the larger COCO dataset, indicating that our model’s generalization capability is slightly inferior to that of PVTv2-B0^26^. However, our model maintains a comparable computational load (FLOPs) while significantly reducing the parameter count by 63.4%. In terms of inference speed, our model achieves an increase of 6 FPS on GPU and an increase of 0.6 FPS on CPU.

In Table 4, the performance of HEViTPose against other models on the MPII test set is similar to that on the validation set, and still achieves competitive performance on fewer model parameter counts and operations compared to state-of-the-art methods, indicating that the HEViTPose model learns more generalisable features. For example, HEViTPose-B achieved a PCKh@0.5 score of 90.7, similar to HRNet-W32^9^, but with a 62.1% reduction in the number of parameters Params and 43.4% reduction in the number of operations FLOPs. Compared with PVTv2-B2^26^, the PCKh@0.5 score of HEViTPose-S is only reduced by 0.3, but the number of parameter Params is reduced by 79.8% and the number of operation FLOPs is reduced by 37.0%. Compared to MobileNetV2^20^, HEViTPose-T has improved PCKh@0.5 score by 2.1 , with 66.5% less Parameter count Params and 17.5% less Operational FLOPs.

**Table 4:** Comparison on the MPII test set(PCKh@0.5). The performance, parameters, and GFLOPs for the pose estimation network are measured w/o considering human detection. All results come from retraining under the same conditions, and none uses any pre-training. We compute the percentages in terms of parameters and GFLOPs reduction between models marked with the same symbol.

Method	Params	FLOPs	Hea	Sho	Elb	Wri	Hip	Kne	Ank	Total
MobileNetV2^20^	9.57M§	2.12G§	97.3	94.5	86.8	80.7	87.3	80.9	74.6	86.6§
Hourglass-52^7^	94.85M	28.67G	97.9	95.8	90.6	86.0	89.1	86.1	81.2	90.0
ResNest-50^47^	35.93M	8.97G	97.9	96.3	90.9	86.0	89.8	86.7	82.1	90.4
ResNet-50^18^	34.0M	7.28G	97.7	95.8	89.8	84.5	89.8	85.7	79.9	89.5
ResNext-50^48^	33.47M	7.48G	97.9	95.8	89.8	84.4	89.5	85.8	80.4	89.5
HRNet-W32^9^	28.02M$\dagger$	9.85G$\dagger$	98.2	96.2	91.3	87.1	90.1	86.8	82.4	90.7
ViTPose-B^49^	90.04M	23.8G	96.7	92.8	85.8	79.5	84.2	78.6	75.0	85.2
PVT-S^22^	28.17M	5.47G	96.9	93.6	86.0	79.8	86.2	79.8	74.4	85.9
Swin-S^24^	54.1M	15.4G	97.5	94.8	88.4	83.0	88.4	83.5	78.4	88.2
EfficientViT-M4^25^	9.87M	5.91G	97.6	95.2	89.4	84.3	89.1	84.5	80.3	89.1
HRFormer-S^23^	7.53M	3.52G	97.8	95.6	90.2	85.8	89.8	85.4	81.0	89.8
PVTv2-B2^26^	29.05M$\dagger$	5.77G$\dagger$	98.0	96.3	90.7	85.9	90.2	86.3	82.4	90.4
HEViTPose-T	3.21M§($\downarrow$66.5%)	1.75G§($\downarrow$17.5%)	97.6	95.1	89.0	83.6	89.1	83.9	79.1	88.7§($\uparrow$2.1)
HEViTPose-S	5.88M$\dagger$($\downarrow$79.8%)	3.64G$\dagger$($\downarrow$37.0%)	97.8	95.9	90.5	86.0	89.7	86.0	81.7	90.1
HEViTPose-B	10.63M$\dagger$($\downarrow$62.1%)	5.58G$\dagger$($\downarrow$43.4%)	98.0	96.1	91.3	86.5	90.2	86.6	83.0	90.7

### COCO keypoints detection

#### Dataset

The COCO dataset^29^ contains more than 200k images and 250k person instances labeled with 17 keypoints. The dataset was divided into train2017, val2017 and test-dev2017 sets with 57k, 5k and 20k images respectively. We train our model on train2017 set, report results of ablation studies on val2017 set and compare with other state-of-the-art methods on test-dev2017 set.

#### Training and testing details

In training and testing, we set the data enhancement pipeline, training strategy, and human pose estimation paradigm the same as MPII. But in order to speed up the training process of the model on the COCO 2017 dataset, we set the batch size to 64 and distributed the model training on 2 NVIDIA RTX4090 GPUs (24GB). In addition, we only applied HEViTPose-B from the family of models found on MPII to COCO, believing that the smaller HEViTPose-S and HEViTPose-T models possess performance in equal proportions to that of the models on MPII.

#### Results on validation set and test set

As shown in Table 5, our HEViTPose-B model achieves high AP values on COCO val2017 with minimal FLOPs and Params. With only 5.58 GFLOPs and 10.63M Params quantities, HEViTPose-B achieves comparable results to HRNet-W32^9^, Swin-S^24^ and PVTv2-B2^26^ . The COCO test-dev2017 in Table 6 demonstrates the effectiveness of the HEViTPose-B model. We observe the comparison of HEViTPose with other models on the COCO validation and test sets, and find that HEViTPose possesses better generalisation ability, and can show greater competitiveness on both the smaller MPII dataset and the larger COCO dataset. For example, compared with Swin-S^24^, a Transformer model with comparable performance, HEViTPose-B has 80.4% fewer Params and 63.8% fewer FLOPs. By looking at Tables 5 and 6, we find that the performance of the Transformer-based model with the support of the large dataset COCO rises significantly over most of the convolution-based models. Our HEViTPose model, which is a hybrid model of convolution and Transformer, has good performance on both smaller dataset MPII and larger dataset COCO.

**Table 5:** Comparison on the COCO val2017 set. The performance, parameters, and GFLOPs for the pose estimation network are measured w/o considering human detection. All results come from retraining under the same conditions, and none uses any pre-training.

Method	Params	FLOPs	AP	AP^50^	AP^75^	AP^M^	AP^L^	AR
ResNet-50^18^	34.0M	7.28G	72.2	92.5	89.3	69.2	76.7	75.4
HRNet-W32^9^	28.02M	9.85G	76.1	93.6	83.6	73.4	80.5	78.9
PVT-S^22^	28.17M	5.47G	70.9	91.5	78.4	68.3	75.2	74.1
ViTPose-B^49^	90.04M	23.8G	73.2	92.5	81.5	71.2	76.6	76.5
HRFormer-S^23^	7.53M	3.52G	73.4	91.6	80.6	70.7	77.9	76.5
HRFormer-B^23^	40.0M	17.35G	77.0	93.6	83.8	74.1	81.4	79.7
Swin-S^24^	54.1M	15.4G	75.6	93.6	83.6	72.9	79.6	78.4
PVTv2-B2^26^	29.05M	5.77G	75.7	93.6	83.5	72.6	80.3	78.5
HEViTPose-B	10.63M	5.58G	75.4	93.6	83.5	72.4	79.6	78.2

**Table 6:** Comparison on the COCO test-dev2017 set. The performance, parameters, and GFLOPs for the pose estimation network are measured w/o considering human detection. All results come from retraining under the same conditions, and none uses any pre-training. We compute the percentages in terms of parameters and GFLOPs reduction between models marked with the same symbol.

Method	Params	FLOPs	AP	AP^50^	AP^75^	AP^M^	AP^L^	AR
Integral^11^	45.0M	11G	67.8	88.2	74.8	63.9	74.0	–
ResNet-50^18^	34.0M	7.28G	69.6	91.0	77.6	66.2	75.5	75.3
PVT-S^22^	28.17M	5.47G	68.9	90.9	77.4	65.7	74.7	74.8
ViTPose-B^49^	90.04M	23.8G	70.9	91.3	79.6	68.4	76.0	76.8
HRFormer-S^23^	7.53M	3.52G	71.0	91.2	79.0	67.7	76.6	76.5
HRFormer-B^23^	40.0M	17.35G	74.0	92.3	82.1	70.6	79.7	79.3
Swin-S^24^	54.1M$\dagger$	15.4G$\dagger$	72.7	92.1	81.5	69.5	78.4	78.3
PVTv2-B2^26^	29.05M	5.77G	72.7	92.1	81.4	69.3	78.4	78.1
HEViTPose-B	10.63M$\dagger$($\downarrow$80.4%)	5.58G$\dagger$($\downarrow$63.8%)	72.6	92.0	80.9	69.2	78.2	78.0

#### Visualisation

**Fig. 8:**
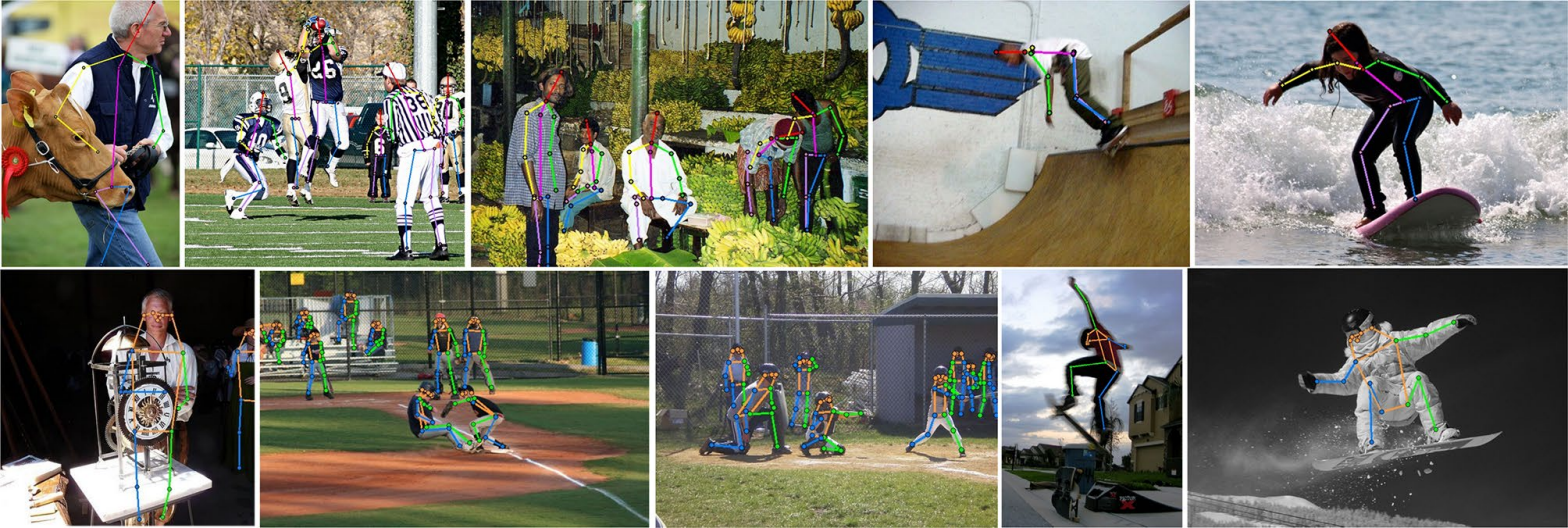
On the MPII (top) and COCO (bottom) data sets, some examples of the prediction results of the HEViTPose network model for human posture include occlusion, multiple people, viewpoint and appearance change.

Figure 8 shows the results of the HPE visualisation of the HEViTPose-B model on the MPII and COCO datasets for each of the complex environments.

### Ablation experiments

In this section, we study the effect of each component on the HEViTPose model on the MPII dataset. All configuration strategies follow the settings in the MPII comparison experiments, including input size $256 \times 256$, batch size 32, etc.

#### Baseline model

In this paper, the algorithmic framework of SimpleBaseline^6^ is adopted as the basis of model design, due to the low feature extraction efficiency of the feature extraction network ResNet-50^18^ adopted in the original paper, the efficient feature extraction network EfficientViT-M4^25^ is introduced, which makes the number of network parameters and computation volume decrease dramatically, and the percentage of decrease reaches 71.0% and 18.8% respectively. However, the accuracy of the model at this time is also reduced by 0.1 percentage points, which is used as the baseline for the model improvement, called SBL-EfficientViT-M4, as shown in Table 7.

**Table 7:** Baseline model(PCKh@0.5).

Method	Params	FLOPs	Total
SBL-ResNet-50^18^	34.0M	7.28G	87.7
SBL-EfficientViT-M4^25^	9.87M	5.91G	87.6

#### Influence of PEOW

We study the effect of different PEOW values based on the baseline SBL-EfficientViT-M4. In order to convert an input image of size $3 \times 256 \times 256$ into features of size $128 \times 64 \times 64$, we satisfy both the image size variation and the PEOW value by controlling the convolution kernel size, convolution type and number of convolutions. As shown in Table 8, proper control of PEOW values can significantly improve the PCKh@0.5 scores of the model while reducing the parameters and computation. Comparing the cases with PEOW of 1, 3, and 7, we find that the model has the highest PCKh@0.5 score of 88.1 when PEOW is 3.

**Table 8:** Influence of PEOW on HEViTPose.

PEOW	(kernel_size, stride)	Params	FLOPs	Total
1	conv(3,2), conv(3, 2)	9.87M	5.91G	87.6
3	conv(7, 4)	9.82M	5.65G	88.1
7	conv(15,8), deconv(4,2)	10.5M	6.29G	85.0

#### Influence of CGSR-MHA

In this section, we observe the variation of the model’s performance on the MPII validation set by controlling various parameters of the CGSR-MHA attention mechanism at each stage of HEViTPose. We used the control variable method to conduct independent ablation experiments on the variables of the number of cascade groups $\{g_1,g_2,g_3\}$, the spatial reduction rate of features within the groups $\{r_1,r_2,r_3\}$, and the number of attentional heads of spatially reduced features within the groups $\{h_1,h_2,h_3\}$, in each stage, respectively. Finally, a set of parameter values that are balanced in terms of model performance, model size, computational efficiency, and training efficiency are determined.

As described in Table 9, when $\{r_1,r_2,r_3\}=\{8,4,2\}$ and $\{h_1,h_2,h_3\}=\{2,4,8\}$ are kept constant, the larger $\{g_1,g_2,g_3\}$, the lower the Params and FLOPs of HEViTPose-B are, and the longer the training time required. When the number of groups $\{g_1,g_2,g_3\} = \{4, 4, 4\}$, HEViTPose-B has the best performance with a PCKh@0.5 score of 89.4.

**Table 9:** Impact of different number of subgroups of the configuration CGSR-MHA on the model for each stage of HEViTPose-B on the MPII validation set (PCKh@0.5). Setting $\{r_1,r_2,r_3\}=\{8,4,2\}$; $\{h_1,h_2,h_3\}=\{2,4,8\}$.

$\{g_1,g_2,g_3\}$	Params	FLOPs	Training time	Total
{2,2,2}	13.72M	5.80G	23h	89.1
{4,4,4}	10.63M	5.58G	26h	89.4
{8,8,8}	9.86M	5.46G	42h	89.0

As described in Table 10, the larger $\{r_1,r_2,r_3\}$ is, the larger the Params and FLOPs of HEViTPose-B are when $\{g_1,g_2,g_3\}=\{4,4,4\}$ and $\{h_1,h_2,h_3\}=\{2,4,8\}$ are kept constant. This is because we use ordinary convolution for in-group feature dimensionality reduction, and the larger the dimensionality reduction ratio, the larger the size of the convolution kernel needed, which will make the Param and FLOPs go up. However, the decrease in the spatial reduction rate will make the training time increase significantly, aggravating the training burden. Therefore, when $\{r_1,r_2,r_3\} = \{8,4,2\}$, the training time of HEViTPose-B is in good balance with other metrics (Table 11).

**Table 10:** Impact of different spatial reduction rates of the configuration CGSR-MHA on the model for each stage of HEViTPose-B on the MPII validation set (PCKh@0.5). Setting $\{g_1,g_2,g_3\}=\{4,4,4\}$; $\{h_1,h_2,h_3\}=\{2,4,8\}.$.

$\{r_1,r_2,r_3\}$	Params	FLOPs	Training time	Total
{4,2,1}	9.87M	5.48G	236h	–
{8,4,2}	10.63 M	5.58G	26 h	89.4
{16,8,4}	13.94 M	5.62G	22 h	89.0

**Table 11:** Impact of different number of attention heads of the configuration CGSR-MHA on the model for each stage of HEViTPose-B on the MPII validation set (PCKh@0.5). Setting $\{g_1,g_2,g_3\}=\{4,4,4\}$; $\{r_1,r_2,r_3\}=\{8,4,2\}$.

$\{h_1,h_2,h_3\}$	Params	FLOPs	Training time	Total
{1,2,4}	10.63M	5.58G	22 h	89.2
{2,4,8}	10.63M	5.58G	26 h	89.4
{4,8,16}	10.63M	5.58G	33 h	89.3

As described in Table 7, when $\{g_1,g_2,g_3\}=\{4,4,4\}$ and $\{r_1,r_2,r_3\}=\{8,4,2\}$ are kept constant, the training time gradually increases as $\{h_1,h_2,h_3\}$ increases. However, the performance increase is not obvious, and the performance of HEViTPose-B reaches the optimum at $\{h_1,h_2,h_3\} = \{2,4,8\}$, at this point, the performance of the model is not increasing by adding more attention heads, which suggests that adding more attention heads will generate computational redundancy.

Since training resources are always limited, we choose a balanced set of parameters for the CGSR-MHA attention mechanism: $\{g_1,g_2,g_3\}=\{4,4,4\}$ $\{r_1,r_2,r_3\}=\{8,4,2\}$, $\{h_1,h_2,h_3\} = \{2,4,8\}$.

#### Design and efficiency-accuracy trade-off of the CGSR-MHA module

The ablation studies above indicate that through the co-optimization of the cascade group number g, spatial reduction rate r, and number of attention heads h, the CGSR-MHA module achieves an effective balance between representational capacity and computational efficiency in human pose estimation. The final configuration embodies a “coarse-to-fine” design strategy. Specifically, aggressive spatial reduction in shallow layers (r = 8) aims to capture global contextual information efficiently at minimal cost, while deeper layers progressively adopt finer reduction rates (r = 4.2), enabling processing of higher-resolution features to recover spatial details for precise keypoint localization. This strategy works synergistically with the high-resolution decoder (Section 3.1) to jointly ensure the final spatial accuracy. Although the cascaded operation in the module (Equation (6)) introduces sequential dependency, its computational overhead is negligible due to the extremely short dependency chain (g = 4) and the fact that the cascaded addition is performed on ultra-low-resolution features (e.g., when r = 8, the feature map size is reduced to 1/64 of the input). Therefore, the combination of short dependency and operations on low-resolution features successfully transforms the theoretical parallel bottleneck into a practically negligible cost. This allows HEViTPose to maintain high precision while achieving the high inference speed reported in Table 3.

#### Ablation of HEViTPose on MPII validation set

In Table 12, we compare the effect of different components on the optimisation of the HEViTPose-B model on the MPII validation set, which finally results in an improvement of 1.8 in the PCKh@0.5 score and a decrease of 5.6% in the computational effort of the model while maintaining the number of parameters. The implementation details of the different methods in the table are as follows:

**Table 12:** Ablation experiments for HEViTPose-B on the MPII validation set (PCKh@0.5).

Method	PEOW = 3	CGSR-MHA	Params	FLOPs	Total
(a)	✗	✗	9.87M	5.91G	87.6
(b)	✓	✗	9.82M	5.65G	88.1
(c)	✓	✓	10.63M	5.58G	89.4

- The baseline model SBL-EfficientViT-M4, where the patch embedding partially follows the OPE configuration of EfficientViT^25^, which corresponds to the case of PEOW of 1 as proposed in this paper. The PCKh@0.5 score of this network is 87.6, the number of parameters is 9.87M, and the amount of operations is 5.91G.
- Adjusting the PEOW to 3 on the basis of method (a) improves the model’s PCKh@0.5 score by 0.5 to 88.1. It also decreases the FLOPs by 4% and the number of parameters slightly.
- Replacing the original CGA^25^ with the CGSR-MHA attention mechanism based on method (b), which further improves the model’s PCKh@0.5 score by 1.3, while keeping the Params and FLOPs at approximate levels.

Moreover, as can be seen from Table 2, the HEViTPose-B model proposed in this paper maintains a comparable level of inference speed on GPUs while maintaining the performance improvement compared to the baseline SBL-EfficientViT-M4.

#### Computational overhead

**Fig. 9:**
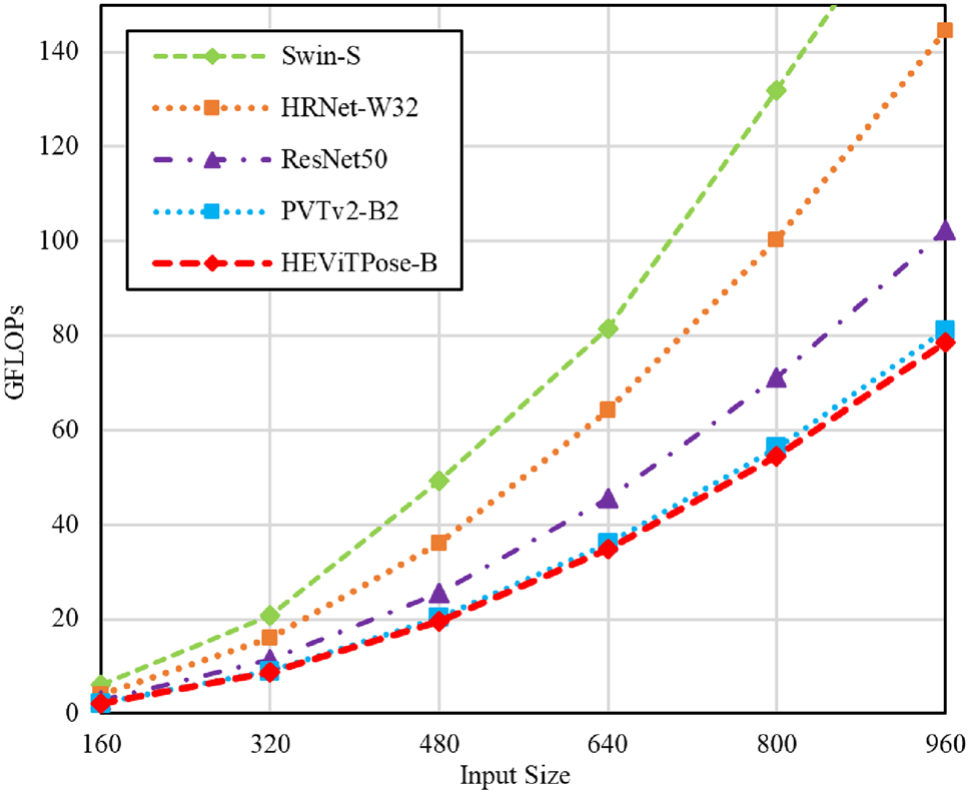
GLOPs of models with different input scales.

In Fig. 9, we analyse the computational overheads of several models that perform best on the MPII and COCO datasets. As the input size increases, the GFLOPs growth rate of the HEViTPose proposed in this paper is much lower than that of the pure convolutional models HRNet-W32^9^, ResNet50^18^, etc., and is also much better than that of the pure Transformer model Swin-S^24^, as well as slightly better than that of PVTv2-B2^26^. This result proves that our HEViTPose is able to effectively solve the problem of computational overhead introduced by the input size increase introduced by the overhead of the attention layer height computation.

## Conclusion

The paper first presents the concept of Patch Embedding Overlap Width (PEOW), which can help readers to further understand the role of Overlapping Patch Embedding (OPE) and provides an effective tool for adjusting the amount of overlap to re-establish local continuity. Then, the paper presents the High-Efficiency Visual Transformer for Human Pose Estimation (HEViTPose), a high-performance and efficient transformer architecture. The main idea is to improve memory efficiency by feature grouping, reduce computational cost by space reduction, and at the same time maintain network performance by retaining a small number of low-dimensional attention heads. Finally, our HEViTPose also benefits from the features containing local continuous information provided by early convolution, and the remote information interaction of Transformers in cascade groups. The combination of convolution and Transformer allows HEViTPose to perform well on both the smaller MPII dataset and the larger COCO dataset. We experimentally validate the effectiveness of HEViTPose on a pose estimation task.

Regarding the model’s generalizability and future work, we provide the following discussion: The core principle of HEViTPose lies in its powerful local detail modeling capacity and efficient structural design. This design principle is not inherently limited to human pose estimation. We believe it can be effectively generalized to other dense prediction tasks that require a balance between accuracy and efficiency, such as animal pose estimation, facial landmark detection, semantic segmentation, and object detection. However, we also acknowledge certain limitations of the model. Firstly, as a heatmap-based method, HEViTPose inherently involves higher computational and memory costs during the output decoding stage, which could become a constraint in extremely resource-constrained scenarios. Secondly, the current architecture is primarily optimized for the topological structure of the human pose; its application to tasks with significantly different topological relationships might require structural adjustments. Therefore, future research will focus on: (i) further optimizing the network architecture to bridge the efficiency gap with regression-based methods in decoding; (ii) exploring hybrid approaches that combine heatmap-based and regression-based supervision to reduce overall complexity and computational cost; and (iii) designing more flexible modules to enhance the model’s adaptability to different task topologies.

## Data Availability

The publicly available datasets, COCO and MPII, used in this study are available at https://cocodataset.org/#download and http://human-pose.mpi-inf.mpg.de/#download, respectively. The source code developed for this study and the processed experimental data supporting the findings are currently hosted in a GitHub repository at https://github.com/T1sweet/HEViTPose. The code will be made publicly available upon acceptance of this manuscript.
